# Near-Zero-Power Temperature Sensing via Tunneling Currents Through Complementary Metal-Oxide-Semiconductor Transistors

**DOI:** 10.1038/s41598-017-04705-6

**Published:** 2017-06-30

**Authors:** Hui Wang, Patrick P. Mercier

**Affiliations:** Department of Electrical & Computer Engineering, University of California, San Diego, La Jolla, California 92093 United States

## Abstract

Temperature sensors are routinely found in devices used to monitor the environment, the human body, industrial equipment, and beyond. In many such applications, the energy available from batteries or the power available from energy harvesters is extremely limited due to limited available volume, and thus the power consumption of sensing should be minimized in order to maximize operational lifetime. Here we present a new method to transduce and digitize temperature at very low power levels. Specifically, two pA current references are generated via small tunneling-current metal-oxide-semiconductor field effect transistors (MOSFETs) that are independent and proportional to temperature, respectively, which are then used to charge digitally-controllable banks of metal-insulator-metal (MIM) capacitors that, via a discrete-time feedback loop that equalizes charging time, digitize temperature directly. The proposed temperature sensor was integrated into a silicon microchip and occupied 0.15 mm^2^ of area. Four tested microchips were measured to consume only 113 pW with a resolution of 0.21 °C and an inaccuracy of ±1.65 °C, which represents a 628× reduction in power compared to prior-art without a significant reduction in performance.

## Introduction

Temperature is an important parameter to measure in a variety of applications such as environmental monitoring, wearable biomedical devices, smart homes, and industrial internet-of-things equipment. Since devices employed in such applications often need to be ultra-small and/or unobtrusive, there is often little room for a battery or energy harvesting source. Thus, the overall power available for such systems is limited, in some cases to below 1 nW^1^, in order to support long system lifetime in a wide variety of applications^2–5^.

To reduce the power of temperature sensing, prior-art has suggested carefully measuring the temperature characteristics of bipolar junction transistors (BJT) integrated on silicon microchips. In such cases, temperature is transduced by comparing the proportional to absolute temperature (PTAT) characteristic of the difference between two base-emitter voltages of a vertical NPN BJT (*ΔV*_*BE*_) with the complementary to absolute temperature (CTAT) characteristic of the base-emitter voltage (*V*_*BE*_), or with a co-integrated constant with temperature (CWT) voltage reference^6–8^. However, biasing BJTs in the forward-active region with sufficiently low noise properties usually demands currents in the nA-μA range that, coupled with supply voltages on the order of a few volts and the power overhead of biasing, control, and analog-to-digital conversion circuits, still exceeds the power demands of ultra-small sensing nodes. To further reduce power consumption, other prior-art has suggested exploiting the temperature-dependency of electron/hole mobility, threshold voltage, and drain currents of MOSFETs^9–11^. Since most modern electronic devices used for amplification, analog-to-digital conversion, digital processing, and wireless telemetry utilize complementary metal-oxide-semiconductor (CMOS) technology, low-cost homogeneous integration of temperature sensing and all other device functionality is possible. Since MOSFETs have several different temperature dependencies, there are many possible ways to create PTAT, CTAT, or CWT references. For example, by connecting the gate, bulk and drain of a p-channel MOSFET together, the characteristic of the drain current with respect to the gate voltage approximates a pn-junction and thus can be employed to detect temperature in a similar way to conventional BJT-based transducers^12^. The temperature-encoded analog signals (currents or voltages) can then be digitized by voltage-, current-, frequency-, or time-to-digital conversion^13–15^. However, all prior-art MOSFET-based techniques still require at least tens of nW of power^16, 17^, and often require external CWT frequency sources for digitization that are not included in the quoted power number. There are thus no current temperature sensing techniques that achieve the sub-nW power consumption necessary to enable next-generation near-zero-power sensing nodes.

Here we present a new temperature sensing technique that relies on complementary temperature dependencies of n- and p-type MOSFETs biased in the subthreshold region, together with CWT tunneling currents and a capacitive charging-time-to-digital feedback architecture that digitizes temperature at 113 pW in a fully monolithically-integrated manner, which represents a 628× reduction in power over prior-art^16^. Specifically, a 2-transitor (2T) subthreshold PTAT voltage reference generator^16^ (VRG) was implemented to serve as the temperature sensing element, while another temperature-stabilized 2T subthreshold VRG^18^ was employed as a CWT reference, replacing conventionally power-hungry band-gap VRGs. The PTAT and CWT analog voltages were then converted to pA-level currents via self-biased current generators based on tunneling effects. Temperature was then digitized by charging digitally-controllable monolithic MIM capacitors with the pA-level currents and matching the charging time between the PTAT and CWT paths via feedback-driven tuning of the MIM capacitors for direct ultra-low-power digital readout.

## Results

Temperature transduction is, at its core, achieved by observing the change in a physical parameter that is temperature-dependent, and comparing that change to a known, ideally CWT, reference. In CMOS circuits, that known reference is typically implemented via a band-gap reference circuit, typically producing 1.25 V (close to the 1.22 eV bandgap of silicon)^19–21^. However, the high required output voltage precludes very low power operation, as low-power CMOS circuits often work at sub-1 V levels. Additionally, most bandgap references require >1 nA^22^, precluding their use for sub-nW systems.

**Figure 1:**
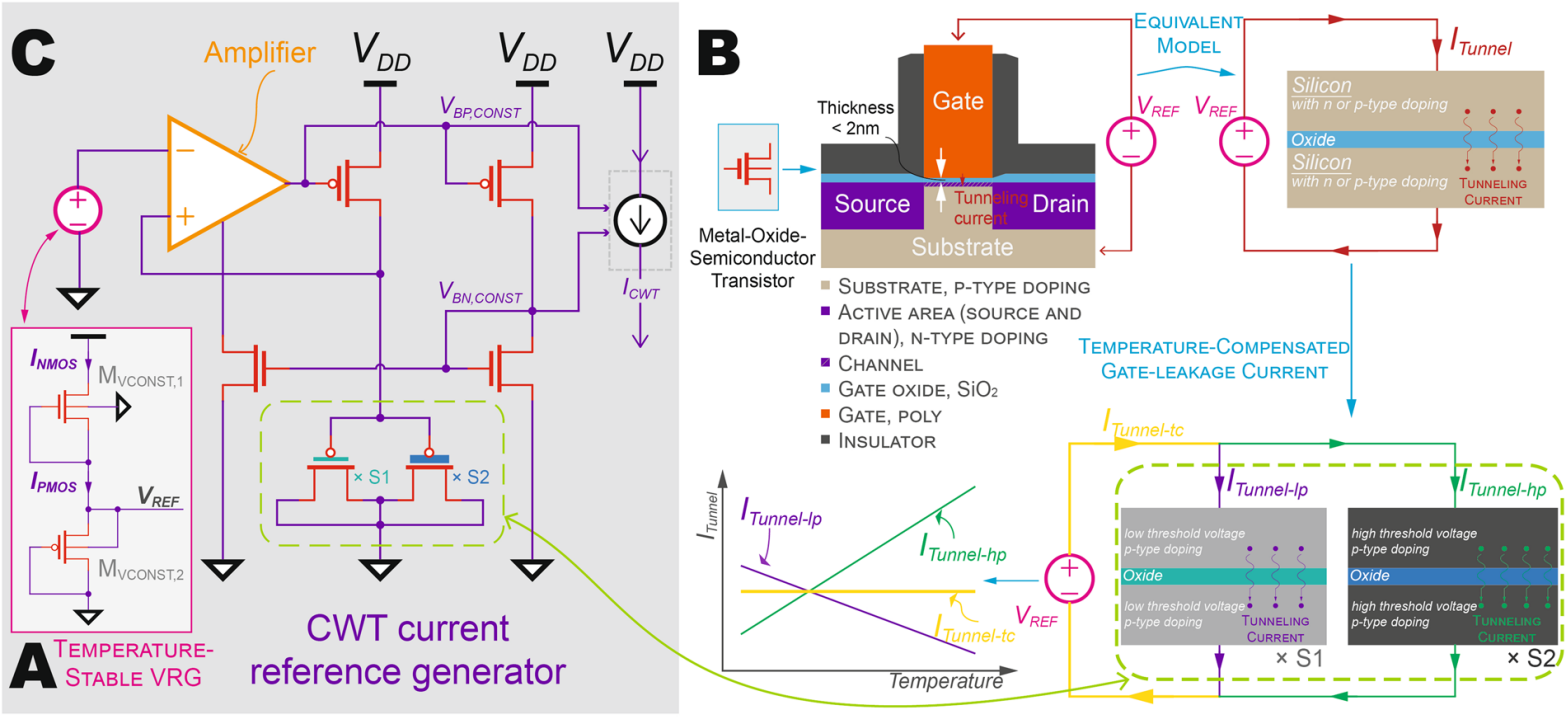
Subthreshold operation and gate-leakage in temperature-stabilized voltage and current reference generators in 65 nm CMOS technology. (**A**) Temperature-stabilized 2 T pW VRG operating in saturated subthreshold region. (**B**) Tunneling current in thin-gate CMOS transistors and temperature-compensated gate-leakage current. (**C**) Monolithic implementation of the pA-level CWT current reference generator.

In this work, we introduce a CWT voltage reference circuit that utilizes only two conventional n- and p-type MOSFETs (NMOS and PMOS) in a two transistor (2T) push-pull arrangement^18^, as shown in Fig. 1A. When biased in the subthreshold or weak-inversion regime (i.e., |*V*_*gs*_ < *V*_*th*_| where *V*_*gs*_ is the gate to source voltage and *V*_*th*_ is the threshold voltage of the transistors), the drain current of each transistor is given by:

1 $${I}_{sub}=\mu {C}_{ox}\frac{W}{L}(n-1){\varphi }_{T}^{2}{e}^{\frac{{V}_{gs}-{V}_{th}}{n{\varphi }_{T}}}(1-{e}^{\frac{-{V}_{ds}}{{\varphi }_{T}}})$$

where *μ* is mobility, *C*_*ox*_ is oxide capacitance, *W* and *L* are the transistor width and length, respectively, *n* is subthreshold slope factor, *ϕ*_*T*_ is thermal voltage, and *V*_*ds*_ is the drain to source voltage. In saturated subthreshold region where *V*_*ds*_ > 4*ϕ*_*T*_, the drain current of the transistor can be calculated by (2):

2 $${I}_{sub}=\mu {C}_{ox}\frac{W}{L}(n-1){\varphi }_{T}^{2}{e}^{\frac{{V}_{gs}-{V}_{th}}{n{\varphi }_{T}}}$$

By equating currents between the NMOS and PMOS, the output reference voltage can be computed, and its temperature sensitivity can be, to a first order, zeroed by appropriate sizing as depicted in Data S1 (Eqs 1–4). Unlike prior work, which required zero-threshold transistors to make a similar transistor arrangement work^23^, the proposed circuit utilized only conventionally-available MOSFETs and was thus implemented at low cost with no additional mask sets required. Implemented in 65 nm CMOS, the VRG generated *V*_*REF*_ = 345 mV with 260.8 ppm/°C measured variation from −20 to 60 °C (Fig. S1A) and power varying from 0.1 pW (−20 °C) to 7 pW (60 °C) (Fig. S1B) over the same temperature range. A similar 2T arrangement, though in this case utilizing two NMOS transistors, was employed to generate a PTAT voltage reference (Fig. S1C). The 2T PTAT VRG generated a PTAT reference, *V*_*REF*, *PTAT*_, with a temperature coefficient of 0.76 mV/°C over the temperature range from −20 to 60 °C (Fig. S1D). The measured power varied from 0.1 pW (−20 °C) to 62.9 pW (60 °C) over the same temperature range (Fig. S1E).

While temperature could be transduced by amplifying the difference between the CWT and PTAT VRGs and digitizing with a voltage-mode ADC, the power overhead of doing so would be large. Instead, we utilized these voltage references to build ultra-low-power current references, whose outputs can be more easily digitized using a novel charging-time-to-digital feedback scheme (described later). Generation of current sources from voltage references typically relies on applying the voltage reference across a CWT resistance via an analog feedback network. However, the pA current levels required here necessitates TΩ resistors given the low VRG voltage levels, which are not conventionally possible to implement on- or off-chip in a small area.

Fortunately, it is possible to generate effectively large resistors in a small on-chip area by exploiting tunneling currents through thin gate oxides available in many modern CMOS processes. For example, the 2 nm SiO_2_ thickness in 65 nm technology facilitates electron tunneling from the conduction band and valence band, and hole tunneling from the valence band, to the point where such gate conduction becomes non-negligible^24–29^. This tunneling current is a function of process parameters (gate oxide thickness and effective mass, barrier height, etc.)^24^ and direct current (DC) bias condition. Transistors doped differently to support, for example, differing threshold voltages, can have opposite temperature coefficients that can be exploited to design CWT tunneling currents. For example, the tunneling current of low threshold (LVT) and standard threshold (SVT) PMOS transistors show opposite temperature dependences^26^. Therefore, the temperature dependence of the gate-leakage current can be minimized by placing appropriately sized LVT and SVT PMOS transistors in parallel with a size ratio of 11:1 and biasing them with a temperature-stabilized reference voltage *V*_*REF*_, therefore enabling temperature-stable pA-level current generation (Fig. 1B).

The monolithically-integrated CWT current reference generator is shown in Fig. 1C, where a self-biased ultra-low-power operational amplifier^18^ provides the feedback path. The total measured power consumption of the CWT current generator was measured to be 3.2 pW. Using a similar topology, a PTAT current generator was implemented employing a 2T PTAT VRG, and consumed 5.8 pW during operation (Fig. S1F).

**Figure 2:**
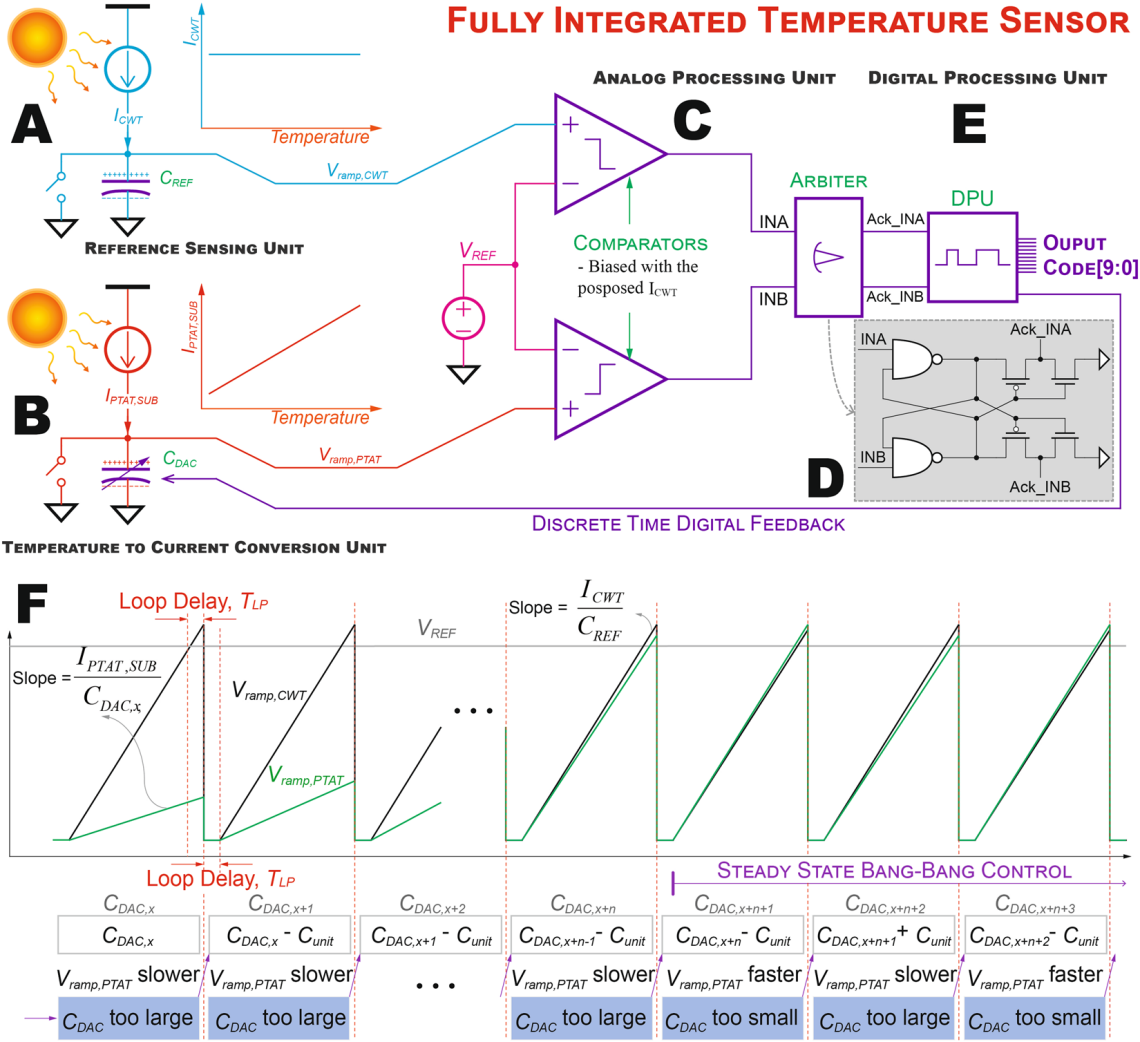
Architecture of the proposed temperature sensor. (**A**) A temperature-stable current source was employed to generate a CWT ramp voltage, *V*_*ramp*_, _*CWT*_, by charging capacitor *C*_*REF*_. (**B**) A PTAT current source was employed as the temperature sensing core by converting temperature to a corresponding current and generated a PTAT ramp voltage, *V*_*ramp*, *PTAT*_, by charging a digitally-controllable bank of capacitors *C*_*DAC*_. (**C**) An analog processing unit consisting of a temperature-stabilized VRG, comparators, and an arbiter was implemented to translate the temperature-encoded analog voltages to digital signals. (**D**) Schematic of the Arbiter. (**E**) A digital processing unit processes the information, controls *C*_*DAC*_, and generates the digital codes corresponding to the ambient temperature. (**F**) An example operation of the DPU illustrates that *C*_*DAC*_ was tuned via discrete time digital feedback to match the rising time of *V* *ramp*, *CWT* in RSU.

The overall architecture of the proposed monolithically-integrated CMOS temperature sensor is shown in Fig. 2. Here, the CWT current reference, *I*_*CWT*_, charges capacitor *C*_*REF*_, generating a ramp voltage *V*_*ramp*, *CWT*_ (Fig. 2A), which serves as the Reference Sensing Unit (RSU). The capacitor is purged (reset) once *V*_*ramp*, *CWT*_ reaches *V*_*REF*_, a temperature-stabilized voltage reference, thus generating an intrinsic temperature-stabilized oscillator. The period of the intrinsic oscillator is

3 $${T}_{OSC}=\frac{{V}_{REF}{C}_{REF}}{{I}_{CWT}}+{T}_{LP}$$

where *T*_*LP*_ is the delay of the loop. At the same time, in the Temperature to Current Conversion Unit (TCCU) shown in Fig. 2B, a PTAT current reference, *I*_*PTAT*, *SUB*_, charges a binary-weighted MIM capacitor, *C*_*DAC*_, generating another ramp voltage, *V*_*ramp*, *PTAT*_. The temperature-encoded voltages are then conditioned by the Analog Processing Unit (APU) (Fig. 2C) where an arbiter (Fig. 2D) was employed to determine which of the two ramp voltages crossed *V*_*REF*_ first. The arbiter output is then used as the input of the Digital Processing Unit (DPU) (Fig. 2E) to determine if *C*_*DAC*_ should be incremented or decremented to match the charging time of *V*_*ramp*, *CWT*_ in the RSU, rendering a 10b output code proportional to temperature via discrete time digital feedback control. Figure 2F shows an example operation of the DPU.

As shown in Fig. S1F, the current from the PTAT reference is given by *I*_*PTAT*_ = *kT* + *I*_*o*_, where *k* is the temperature coefficient, *T* is the absolute temperature, and *I*_*o*_ represents an offset. Therefore, at steady state

4 $$\frac{{I}_{CWT}}{{C}_{REF}}=\frac{{I}_{PTAT}}{{C}_{DAC}}=\frac{kT+{I}_{o}}{{C}_{DAC}}.$$

Across the temperature range from *T*_*min*_ to *T*_*max*_, the minimum and maximum required *C*_*DAC*_ can be calculated by (5) and (6), respectively,

5 $${C}_{\min }=(k{T}_{\min }+{I}_{o})\frac{{C}_{REF}}{{I}_{CWT}},$$

6 $${C}_{\max }=(k{T}_{\max }+{I}_{o})\frac{{C}_{REF}}{{I}_{CWT}}.$$

The temperature-to-digital conversion resolution *T*_*LSB*_, therefore, can be calculated by

7 $${T}_{LSB}=\frac{{T}_{\max }-{T}_{\min }}{{C}_{\max }-{C}_{\min }}=\frac{1}{k}\frac{{I}_{CWT}}{{C}_{REF}}$$

**Figure 3:**
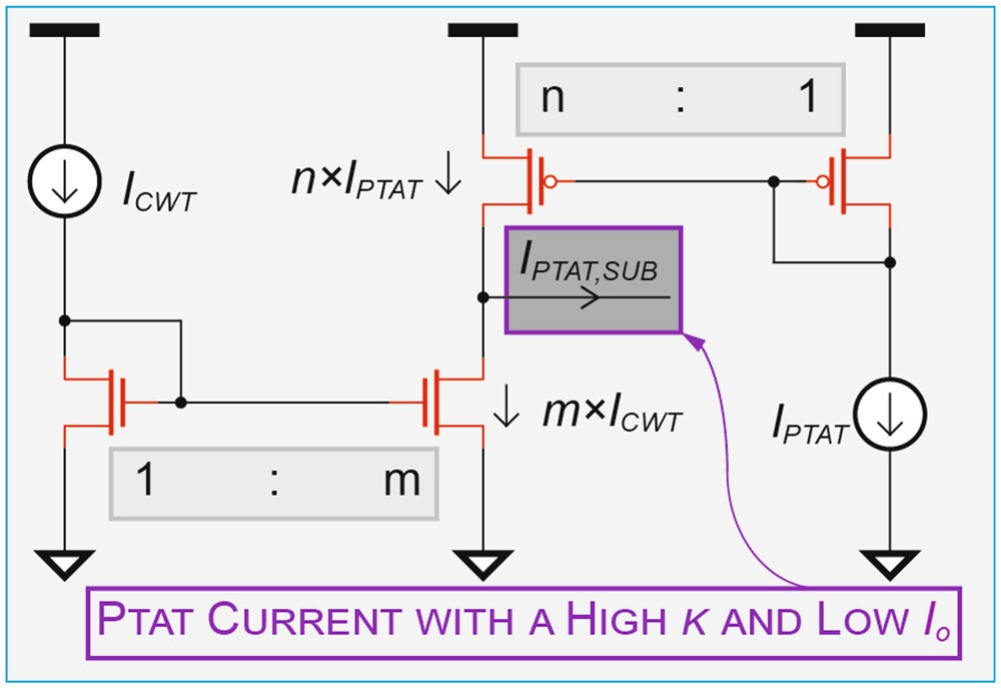
Implementation of the current subtractor employed to increase temperature conversion resolution by increasing the effective temperature coefficient *k*, where m = 1 and n = 3.

As shown in (5) and (6), the area of the capacitor *C*_*DAC*_ (which can dominate the chip size) is proportional to *I*_*o*_, while (7) indicates that the achievable temperature-to-digital conversion resolution is inversely proportional to *k*. Thus, to achieve a large resolution in small area, a current subtractor was employed (Fig. 3), whereby n × *I*_*PTAT*_ is subtracted from m × *I*_*CWT*_ to generate *I*_*PTAT*, *SUB*_, effectively multiplying the temperature coefficient (and therefore resolution) by n (n = 3 in this implementation and is trimmable), while reducing the required capacitor *C*_*DAC*_ area by a factor of n-m × *I*_*CWT*_/*I*_*o*_ (2.2 in this implementation).

**Figure 4:**
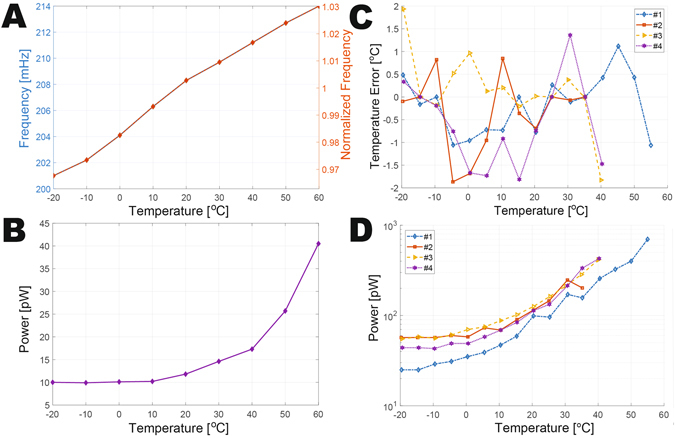
Experimental results of the intrinsic oscillator and temperature sensor. (**A**) Measured oscillation frequency of the intrinsic oscillator across temperature at V_DD_ = 0.5 V. (**B**) Measured power of the intrinsic oscillator across temperature at V_DD_ = 0.5 V. (**C**) Measured temperature error across temperature at V_DD_ = 0.5 V. (**D**) Measured power of the temperature sensor across temperature at V_DD_ = 0.5 V.

The proposed temperature sensor was fabricated in a standard 65 nm CMOS, and occupied 0.15 mm^2^. The intrinsic oscillator alone occupied an area of 0.038 mm^2^, and, as shown in Fig. 4A, oscillated at 0.208 Hz and consumed 11.8 pW at 20 °C (Fig. 4B). The oscillator achieved a temperature coefficient of 772 ppm/°C (Fig. 4A) and line regulation was measured to be 6%/V from 0.4 V to 1.0 V (Fig. S2A).

Four samples of the temperature sensor were tested in a temperature-controlled oven. At 0.5 V, the temperature sensor output codes were measured to be stable across the temperature range (a representative transient waveform is shown in Fig. S2B). The accuracy of temperature sensing was measured by ramping ambient air temperature at a rate of 0.2 °C/minute from −20 to 40 °C, and comparing the digital sensor output to the readings of a proximal platinum resistance thermometer. As shown in Fig. 4C, the temperature sensors achieved a worst-case inaccuracy of ±1.93 °C after a second order polynomial fit to their average characteristic. Measured from 1500 consecutive conversions, the temperature sensing resolution was 0.21 °C at 20 °C.

**Figure 5:**
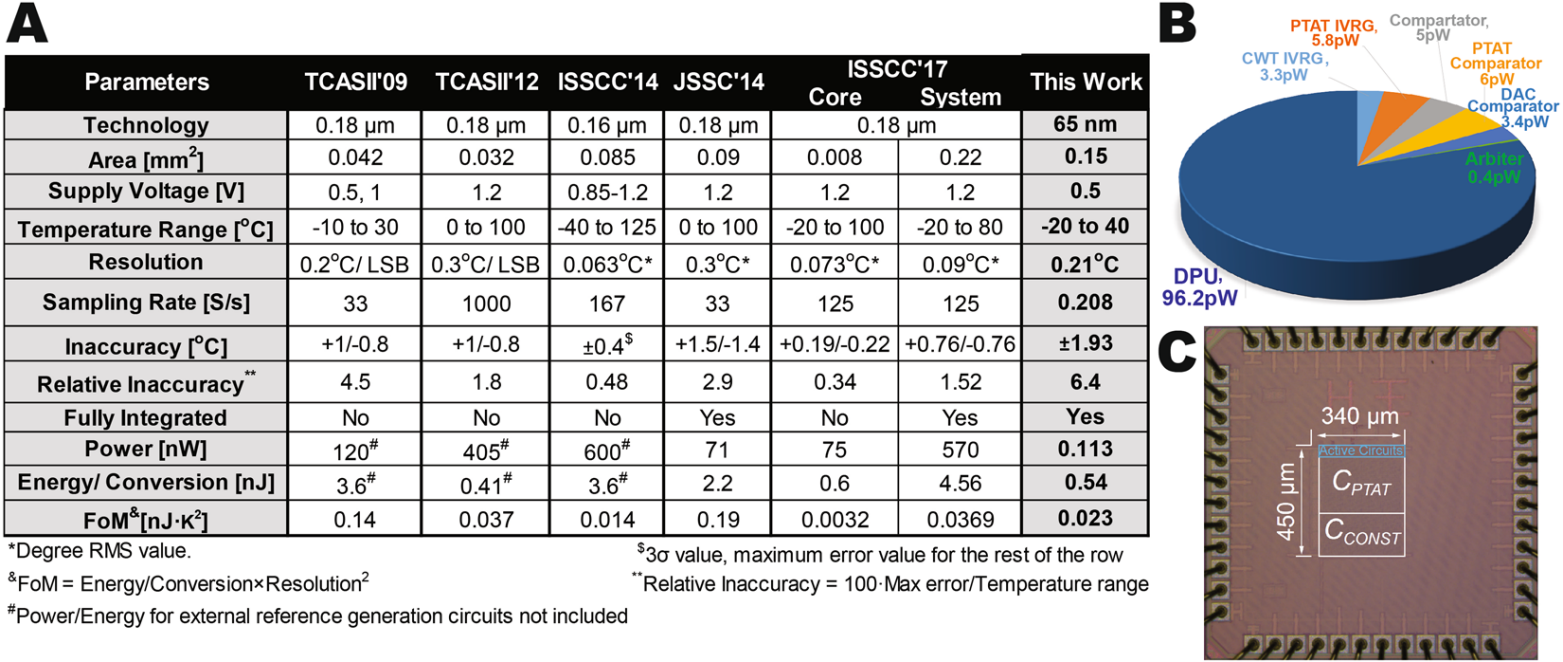
Summarized experimental results of the temperature sensor and the fabricated temperature sensor chip. (**A**) Comparison with prior-art. (**B**) Power breakdown of the proposed temperature sensor. (**C**) Micrograph of the proposed fully integrated temperature sensor.

At 0.5 V, the four temperature sensor samples consumed 113 pW at 20 °C (including the fully on-chip RSU, APU, and DPU), which improves state-of-the-art^16^ by 628× as indicated in the comparison table in Fig. 5A ^12, 14, 17, 30, 31^. The power of the DPU, which consists of digital control logic, counters, level shifters, etc., dominates the system-level power consumption, as indicated by the power breakdown in Fig. 5B. The temperature sensor required 4.8 s of conversion time, resulting in 540 pJ/conversion, which is >4× lower than prior fully-integrated temperature sensors (including the energy of all reference generators). It should be noted that while prior-art designs can in principal be duty-cycled to achieve low average power given similar energy efficiency metrics, the power of always-on reference generators and oscillators are difficult to scale, and power gating transistors have finite on resistance and off currents, limiting the ability to simply scale prior-art architectures down to sub-nW levels without significant re-design efforts. A die photograph of the fully-integrated temperature sensor is shown in Fig. 5C.

## Discussion

The sensor described in this work enables transduction and digitization of temperature at 628× lower power than prior-art without a significant reduction in sensing accuracy. By combining the generation of CWT and PTAT voltages via subthreshold-biased 2T circuits with tunneling-current-based CWT resistances to generate CWT and PTAT currents, and using these currents in a feedback circuit that normalizes charging time via a digitally-controlled capacitor bank, temperature was directly digitized at ultra-low-power. With a relaxation oscillator intrinsically built into the proposed architecture, no external references, biases, clocks, or any other components are required for temperature-to-digital transduction. The proposed sensor enables a new class of devices that can monitor their environments with nearly zero power, enabling ultra-long battery life, or energy harvesting from low-power sources towards energy-autonomous operation. While measurements across the four chips presented in this paper gives an idea of the accuracy of the proposed temperature sensor in the presence of process variation, more die-to-die, wafer-to-wafer, and lot-to-lot measurements would be needed in future work to validate accuracy for volume manufacturing.

## Methods

### Integrated circuit fabrication

The prototype chip was fabricated in a 65 nm 1P9M (1 poly layer and 9 metal layers) commercial technology (Fig. 4G).

### Monolithic MIM capacitors

As shown in Fig. 4G, MIM capacitors *C*_*REF*_ and *C*_*DAC*_ were sized to be 4.6 pF and 9.7 pF, respectively.

### Ultra-low-leakage CMOS switch design

Since the circuit operates at tens of pA, temperature-dependent leakage across reset switches can inadvertently discharge *C*_*REF*_ and *C*_*DAC*_, significantly affecting linearity and temperature stability. Thus, an ultra-low leakage switch was employed, whereby two NMOS transistors were connected in series, and the source node was actively driven to the drain potential via a 1.8 pW operational-amplifier (op-amp) and a gate-boosted control switch (Fig. S3A). Simulations reveal a 50× improvement of leakage variation across the temperature range, to less than 21.5 ± 0.5 fA. To maximize the linearity of the capacitive digital-to-analog converter (DAC) at low voltage, binary-weighted switches were designed with dynamic threshold and boosted gate control, improving linearity by 13.2× (Fig. S3B).

### Measurement bench

The prototype chip was wire bonded with a 48-pin quad-flat no-leads (QFN-48) package and mounted on a printed circuit board fabricated with an FR-4 substrate. The traces on the PCB were shielded and solder mask between the PCB traces was removed to minimize the leakage. Sub-Miniature version A (SMA) connectors were mounted on the PCB for power supplies and digital readout. The PCB was placed in a TestEquity 106 temperature chamber which controlled the environmental temperature of the chip in the measurement. A platinum resistance temperature device (Thomas Traceable Resistance Temperature Detectors Platinum Thermometer) was placed next to the chip and used as a temperature reference. An Opal Kelly XEM6310 field-programmable gate array (FPGA) was employed to read the digital output of the temperature sensor and transferred the data to Matlab. All power consumption measurement in this work were taken with a Keithley 6430 sub-fA SourceMeter with high-isolation shielded triaxial cables.

### Data availability

All relevant data is available from the authors on request.

## Electronic supplementary material

Dataset 1
