# Supplementary material for: Near-Zero-Power Temperature Sensing via Tunneling Currents Through Complementary Metal-Oxide-Semiconductor Transistors
Source: Sci Rep. 2017 Jun 30;7:4427. doi: 10.1038/s41598-017-04705-6 (PMC5493670; doi:10.1038/s41598-017-04705-6)
Supplement: Supplementary file 1 — Dataset 1 [file 41598_2017_4705_MOESM1_ESM.doc]

### Near-Zero-Power Temperature Sensing via Tunneling Currents Through Complementary Metal-Oxide-Semiconductor Transistors

### Hui Wang^a^, Patrick P. Mercier*^a^

^a^Department of Electrical & Computer Engineering,

University of California, San Diego, La Jolla, California 92093

*E-mail: [pmercier@ucsd.edu](mailto:pmercier@ucsd.edu)

(1)

$I_{NMOS}=I_{PMOS}.$ (2)

(3)

$\frac{\partial V_{REF}}{\partial T}=0\Rightarrow(\frac{W_{1}}{L_{1}})/(\frac{W_{2}}{L_{2}})=\frac{\mu_{2}C_{ox2}(n_{2}-1)}{\mu_{1}C_{ox1}(n_{1}-1)}e^{\frac{q(n_{2}C_{V_{th1}}-n_{1}C_{V_{th2}})}{n_{1}n_{2}k}}.$ (4)

***Data S1. Sizing of the NMOS and PMOS transistors in the temperature-stabilized 2T pW VRG operating in saturated subthreshold region.***


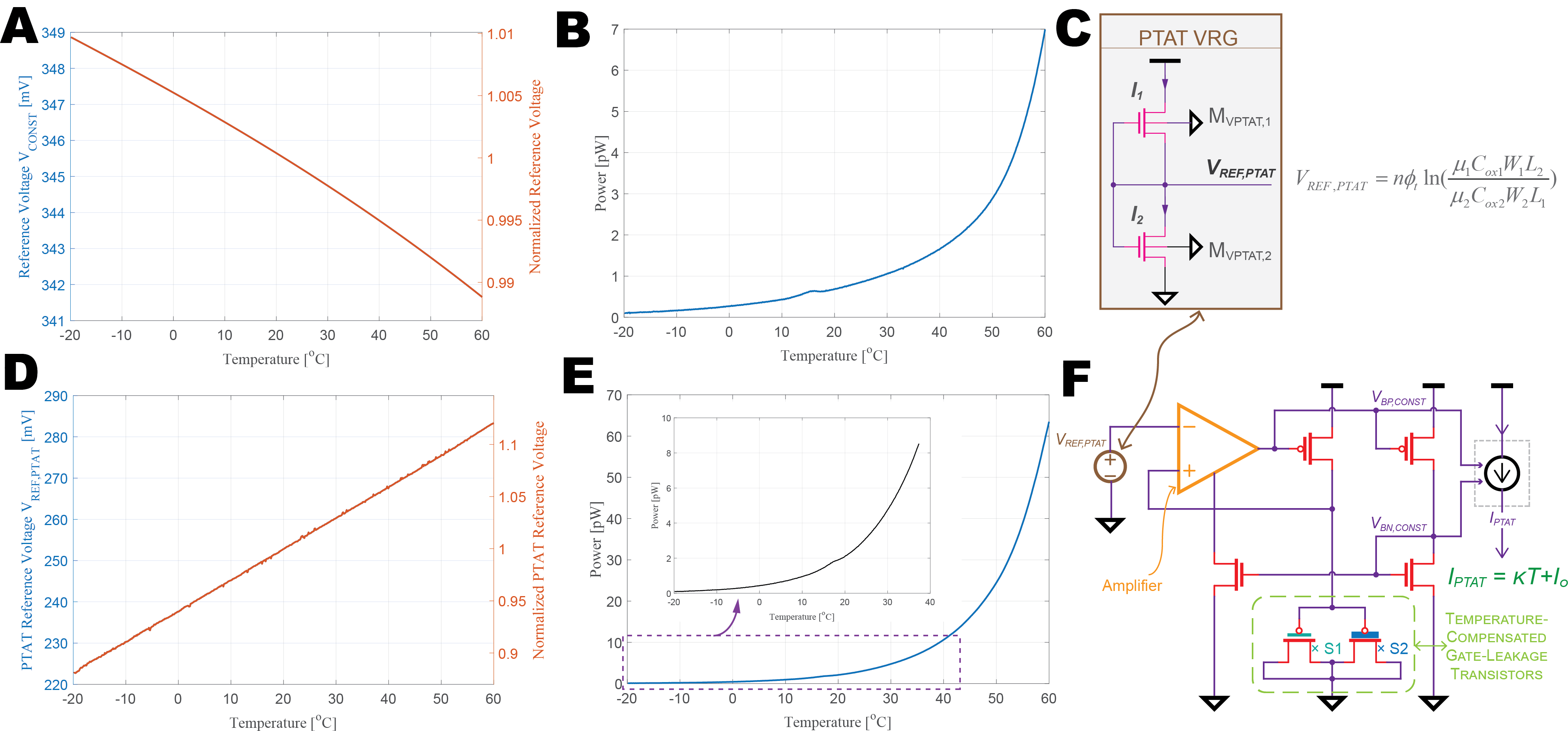


***Figure S1. 2T Temperature-stabilized and PTAT VRGs experimental results and implementation of the PTAT voltage and current reference generators. (A) Experimental results show that V_REF_ achieved a temperature coefficient of 260.8 ppm/^o^C over the temperature range from -20 to 60^o^C. (B) The power of the 2T temperature-stabile VRG varied from 0.1 pW (-20^o^C) to 7 pW (60^o^C) over the same temperature range. (C) The PTAT VRG was implemented with a 2T structure employing two regular NMOS transistors operating in saturated subthreshold region without any special mask sets required. (D) Experimental results show that the PTAT reference V_REF,PTAT_ achieved a temperature coefficient of 0.76 mV/^o^C over the temperature range from -20 to 60^o^C. (E) The measured power of the 2T PTAT VRG varied from 0.1 pW (-20^o^C) to 62.9 pW (60^o^C) over the same temperature range. (F) Monolithic implementation of the PTAT current reference generator employed the 2T VRG and temperature compensated gate-leakage transistors.***


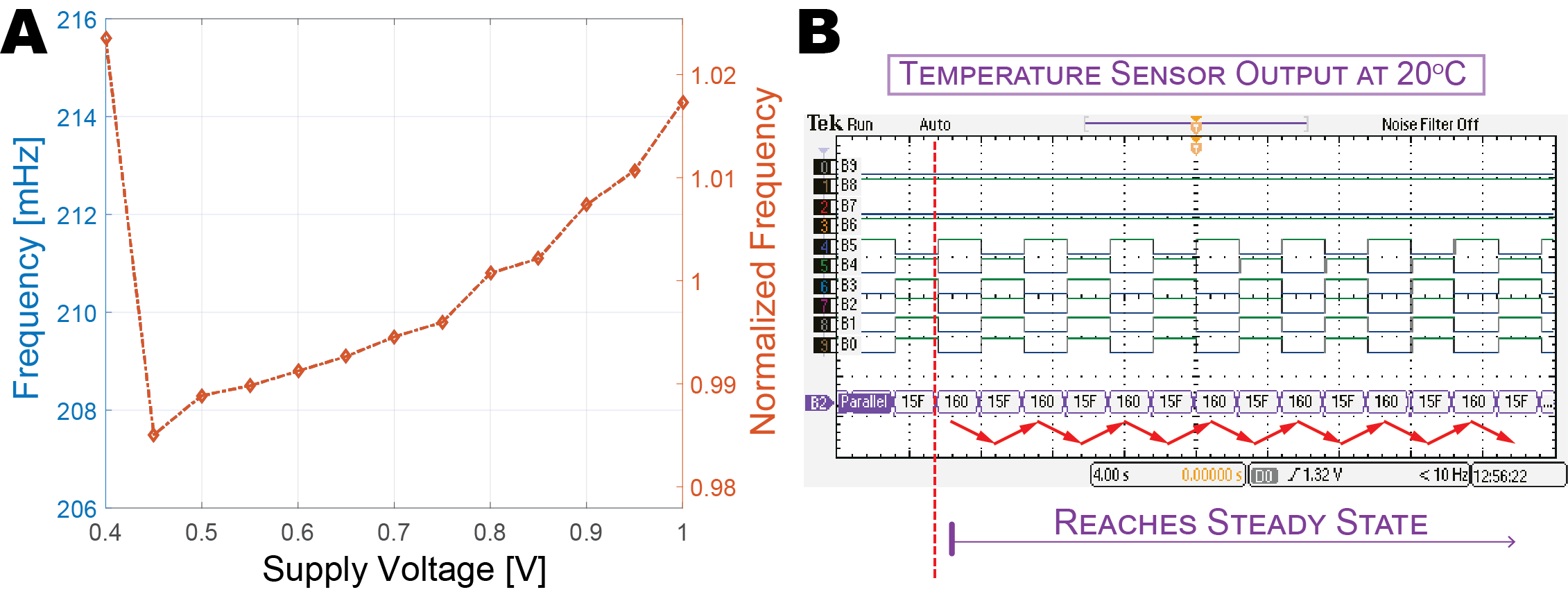


***Figure S2. Experimental results of the intrinsic oscillator and an experimental output of the temperature sensor. (A) Measured oscillation frequency of the intrinsic oscillator over the supply range from 0.4 to 1 V. (B) An example experimental operation of the proposed temperature sensor.***


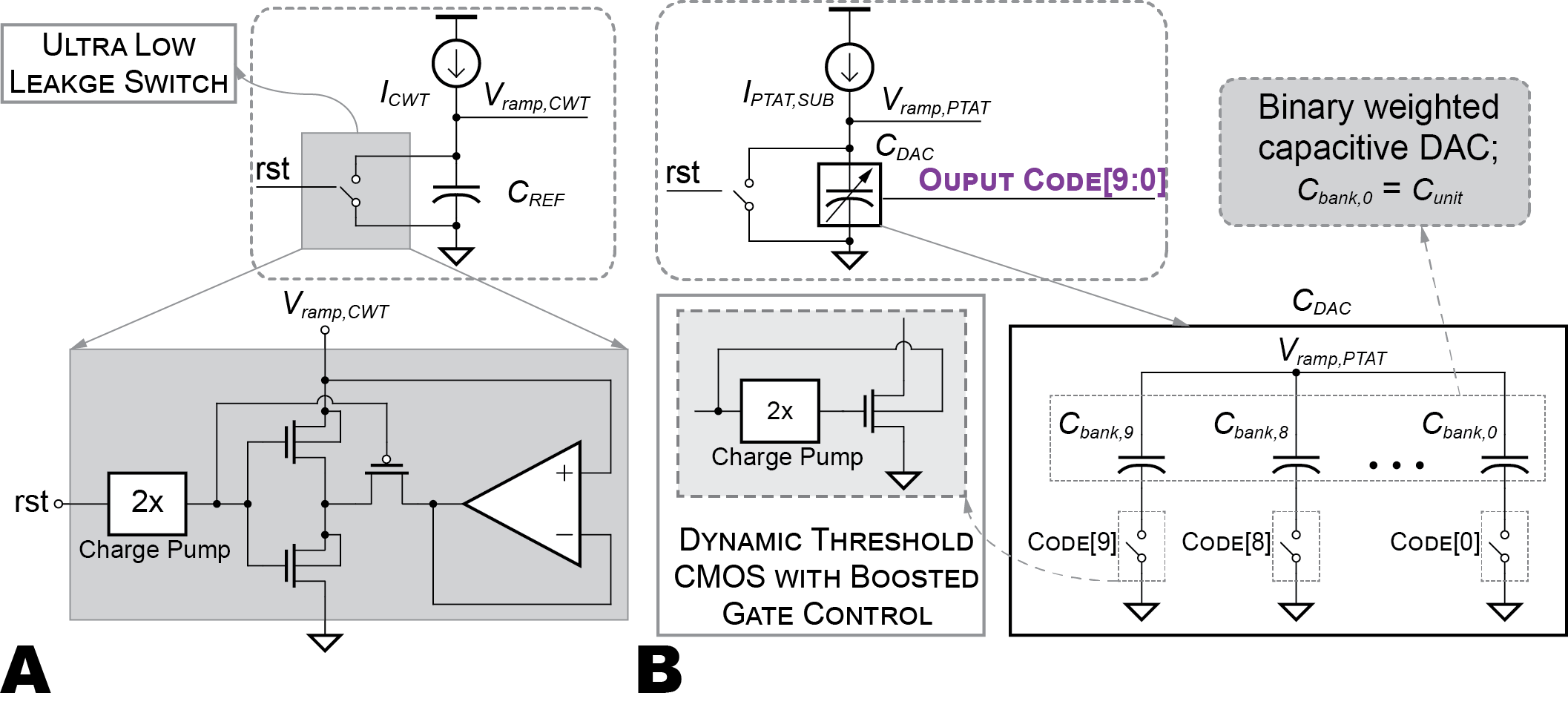


***Figure S3. Ultra-low-leakage CMOS switches implementation. (A) The monolithic implementation of the ultra-low-leakage switch in RSU. (B) The implementation of the ultra-low leakage switch and capacitor bank in TCCU.***

**
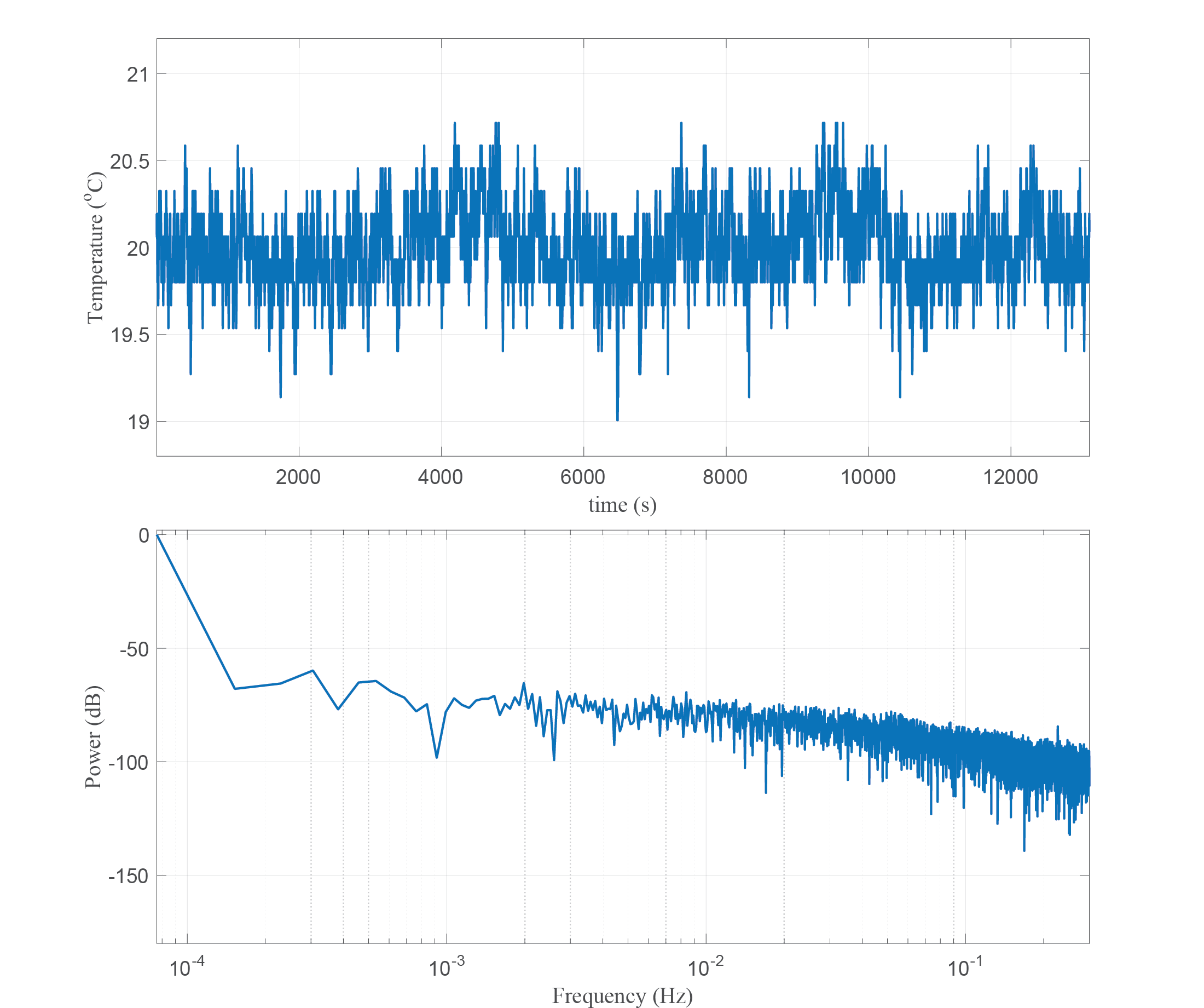
**

***Figure S4. Long-term stability of the proposed temperature sensor. The temperature sensor was measured for 3.7 hours at 20 ^o^C to test its stability over time. The temperature sensor showed a variation of less than +0.71/-0.9 ^o^C, which is just slightly higher than the control tolerance of the temperature chamber itself.***
